# The Assessment of Attitudes of Students at Medical Schools towards Psychiatry and Psychiatric Patients—A Cross-Sectional Online Survey

**DOI:** 10.3390/ijerph18094425

**Published:** 2021-04-21

**Authors:** Mateusz Babicki, Krzysztof Kowalski, Bogna Bogudzińska, Patryk Piotrowski

**Affiliations:** 1 Department of Family Medicine, Wroclaw Medical University, 51-141 Wroclaw, Poland; 2 Students’ Scientific Group at the Faculty of Psychiatry, Wroclaw Medical University, 50-367 Wroclaw, Poland; krzysztof.kowalski.umed@gmail.com (K.K.); bognaa95@gmail.com (B.B.); 3 Section of Epidemiology and Social Psychiatry, Department and Clinic of Psychiatry, Wroclaw Medical University, 50-367 Wroclaw, Poland; patryk.piotrowski@umed.wroc.pl

**Keywords:** mental health, stigmatisation, MICA-2

## Abstract

The aim of the study was the assessment of the level of stigmatisation of psychiatric patients and psychiatry as a field of study by students at medical schools in Poland and the comparison of students’ attitudes over the years. The study was conducted based on a proprietary questionnaire assessing the stage of tertiary education, sociodemographic status, and MICA-2 psychometric tool that is used for assessing both the attitudes of students at medical schools towards psychiatry as a field of study and patients with mental health disorders. According to the MICA score, those who have higher scores have more negative attitudes towards psychiatry. The survey consisted of two rounds at an interval of 3 years. Results. The first-round survey, conducted in 2017, involved 480 students. The second-round survey, conducted in 2020, involved 573 students. In both cases, women constituted the vast majority of respondents. Women, as well as medical major students, achieved significantly lower scores than men *p* < 0.001. The said relationship was also observed for individual experience with mental illness. There was no correlation between the MICA-2 total score and the psychiatry course completion—*p* = 0.105. However, the levels of stigmatisation are still high. The implementation of educational methods to improve the perception of psychiatric patients by students at medical schools should be taken into consideration. An increase in direct student–patient contact, for example, by means of intensive elective classes, could be beneficial.

## 1. Introduction

According to the report published in 2011 by the European College of Neuropsychopharmacology and the European Brain Council, which is a result of a three-year study, 38.2% of EU citizens per year suffered from a mental health disorder. This result makes mental health disorders one of the greatest challenges of modern medicine [1]. The patients with mental disorders have to face not only their health problem but also their perception by society [2]. Stigmatisation is a process of stigma creation and leads to the ostracism of individuals from various parts of social life [3]. This may be due to citizens’ lack of knowledge, prejudice, and discriminatory behaviour [4]. The stigmatisation process can be a real obstacle to patient treatment [5].

The stigma attached to mental disorders causes psychiatry to be perceived as less attractive, which is related to a decline in interest in psychiatry as a career path chosen by medical major students. A similar phenomenon is observed not only in Poland but also worldwide [6,7]. According to studies involving students of medicine, they consider patients with mental disorders to be unpredictable and dangerous. Moreover, 4–21% of students at medical schools consider schizophrenia to be an incurable disease [8]. In a survey conducted in 20 countries, only 4.5% of respondents chose psychiatry as their potential career path [9].

According to studies, the ratio of the number of psychiatrists in Poland is 9.02 per 100,000 citizens. Poland ranks penultimately in the European Union. In the best functioning health care systems in Europe, i.e., Germany, Greece, France and the Netherlands, this ratio ranges between 22.89and 27.33 [1].

Due to a significant shortage in psychiatrists, the authors of scientific reports suggest that appropriate strategies should be implemented to increase the attractiveness of psychiatry and thus the interest in psychiatry as a field of study. This would enable meeting the needs of the health care system in the future [10], in particular, that the problem of stigma is common among medical students. Although they have advanced medical knowledge, the research suggested that they keep a great distance from patients with mental disorders. The above-described relationship was confirmed by a study evaluating the views of medical students on the aetiology of diagnosis and prognosis in schizophrenia, conducted in a group of 381 people. Observations on another 419 students confirmed that they have difficulties in direct contact with patients; they focus mainly on pharmacotherapy, avoiding psychological aspects. The psychiatric curricula for medical students should include the greater integration of psychological and pharmacological aspects. [8]

Tackling stigmatising attitudes among students of medicine is important for effective medical interventions in the future [11]. There are several tools that assess the level of stigmatisation. The MICA Scale is the most reliable and universal tool [12]. This is a short, fully autonomous questionnaire used for assessing health care professionals’ attitudes towards psychiatric patients and psychiatry as a field of study. MICA-2 is a version designed for students at medical schools.

This study aims to assess the level of stigmatisation of psychiatric patients and psychiatry as a field of study by students at medical schools in Poland and the comparison of students’ attitudes over the years. As part of the study, the following hypotheses were proposed: (1) Students after completing the course in psychiatry show a lower level of stigmatisation. (2) Women show more favourable attitudes towards psychiatric diseases than men. (3) Medical students show a lower level of stigma than paramedical students. (4) Own experiences with mental disorders affect the level of stigmatisation.

## 2. Materials and Methods

### 2.1. Methods

The survey was conducted using a proprietary questionnaire distributed online through a social network. The survey was fully anonymous and voluntary. Participants were informed about the objectives, duration, and methodology of the survey before they were allowed to take part in the survey. After the respondents were familiarised with the information, they gave their informed consent to be surveyed. The respondents were given the opportunity to opt out at any stage of the survey without giving any reason. Finally, the respondents were given contact details of the authors of the survey.

The survey consisted of two rounds. The first-round survey concerned the stigmatisation process of students at medical schools in Poland in 2017. The survey was repeated in 2020.

The presented study was approved by the Bioethics Committee of the Wroclaw Medical University, and it was conducted in accordance with the Declaration of Helsinki.

The questionnaire consisted of two parts. The first part addressed questions assessing sociodemographic status, i.e., age, sex, and place of residence, as well as questions concerning the stage of tertiary education, including the year of study, taking psychiatry classes, and having personal experience with mental illness. The second part of the questionnaire was a standardised psychometric tool, MICA-2. The Mental Illness Clinicians’ Attitude Scale (MICA-2) is a 16-item psychometric tool designed to assess the attitudes of students at medical schools towards both psychiatry as a field of study and patients with mental health disorders. The questionnaire is based on a 6-point Likert scale [13]. Questions 3, 9, 10, 11, 12, and 16 are scored as follows: I strongly agree = 1 point; I agree = 2 points; I rather agree = 3 points; I rather disagree = 4 points; I disagree = 5 points; I strongly disagree = 6 points. Other questions are scored inversely [14].

The analysis of the tool is based on the overall assessment of the number of points scored by the participants. The score range is between 16 and 96 points. High scores indicate stigmatising attitudes towards both psychiatry as a field of study and psychiatric patients [13]. The Mica-2 scale shows high internal consistency and reliability [12]. However, the questionnaire has not been yet validated in Polish conditions.

The tool can also be interpreted at the level of individual questions. It should also be noted that the individual questions were divided into five thematic groups, as proposed by Gabbidon et al. [13]:

(a) Views concerning mental illness and mental health care (questions 3, 10, 12, 16);
(b) Knowledge of mental illness (questions 1, 2, 5, 6);
(c) Willingness to disclose one’s own mental illness (questions 4, 7);
(d) Distinction between physical and mental health (questions 8, 13, 14, 15);
(e) Medical care for patients with mental illness (questions 9, 11).

### 2.2. Statistical Analysis

The statistical analysis was performed using the Statistica 13 software, (Cracow, Poland, StatSoft.) The variables were qualitative (and dichotomous), interval, as well as ordinal.

A chi-squared test was used for determining the relationships between the compared ordinal variables. Depending on the strength in two-way tables, the Yates’s chi-squared test and a detailed F-test were also used. For variables based on interval scales, basic descriptive statistics were determined. The normality of distributions of those variables was assessed using three different statistical tests: the Kolmogorov–Smirnov test, Lilliefors test, and Shapiro–Wilk test at the significance level of *p* = 0.05. The homogeneity of variance was assessed using the Levene’s test and Brown–Forsy the test at the assumed significance level of *p* = 0.05. The statistical significance of differences between mean values was assessed using the nonparametric Mann–Whitney U test or the Kruskal–Wallis test. An analysis of covariance (ANCOVA) was then performed to assess the differences between two rounds of research while taking into account the potential confounding factors (sex, major, year of study, psychiatry classes undertaken, and mental health problems)

A confidence level of α = 0.05 was assumed in all tests assessing the statistical significance of differences between means.

## 3. Results

### 3.1. Participants

A detailed profile of the study group is shown in Table 1.

A total of 1053 respondents (representing students at medical schools and universities from all over Poland) were surveyed. Among them, 645 (61%) were students of medicine, while 408 (39%) were students of other majors at medical schools (i.e., dentistry, nursing, obstetrics, and medical rescue). The mean age of the respondents was 22.91 years (min. 18, max. 48; SD 3.13). The first 2017 round survey involved 480 participants, while the second 2020 round survey involved 573 participants. The mean age was 23.40 (±2.5) and 22.51 (±3.51), respectively.

### 3.2. The Analysis of the MICA-2 Questionnaire

In the survey, the mean MICA-2 score among the respondents was 41.05 (min. 20, max. 80; SD 8.09) points. Women obtained significantly lower values than men (40.39 ± 7.59 vs. 43.94 ± 9.44)—*p* < 0.001. There were no statistically significant differences between the year of studies and the mean MICA-2 score (*p* = 0.071).

Individual experience with one’s own mental illness (40.48 ± 7.37) and that in immediate family members and friends (40.03 ± 8.34) was related to significantly lower MICA-2 scale scores compared to the lack of such experience (44.10 ± 7.65; *p* < 0.001). Completion of a course in psychiatry did not have any statistically significant effect on the overall score of the survey (41.35 ± 8.28 vs. 40.76 ± 7.90 (*p* = 0.105).

Students of medicine achieved a lower mean score on the MICA-2 scale compared to students studying other majors at medical schools (40.09 ± 8.38 vs. 42.56 ± 7.36; *p* < 0.001).

Table 2 shows a detailed comparison of responses to each question.

As regards future physicians, both women (*p* < 0.001) and men (*p* = 0.002) achieved significantly lower scores than their co-workers studying other majors at medical schools. In both cases, one’s own experience with mental illness affects the perception of psychiatric patients in a significant way (*p* < 0.001). A detailed comparison is shown in Table 3.

According to the analysis of individual questions addressed in MICA-2, as many as one out of three surveyed students admit that they studied psychiatry only because they had an exam, and they show little interest in psychiatry as a field of study. At the same time, 97% of the respondents consider psychiatry to be an equal field of study as other branches of medicine. The survey shows that 3% of future medical workers believe that a psychiatrist is not a “real doctor.” However, as many as 71% of the respondents believe that psychiatrists know more about their patients’ lives than their family members. More than half of the respondents believe that psychiatric patients often pose a threat to other people, and 59% of students disagree that society should not be protected from seriously mentally ill patients. There is a noticeably high fear of disclosing one’s own mental illness, and nearly 31% of the respondents claim that if they suffered from mental illness, they would never tell any of their friends about it. A total of 33% of the surveyed students were reluctant to continue professional cooperation with a person suffering from a mental health disorder. The exact distribution of responses addressed in MICA-2 is shown in Table 4.

As regards most aspects addressed in the MICA-2 questionnaire, women were significantly more likely to express a positive judgment than men—*p* < 0.05. A similar relationship was observed when assessing the likelihood of using the word “nut” and “madman” towards a mentally ill person, where 93% of female medical workers and 82% of male medical workers would not express such an opinion—*p* < 0.001. A total of 97% of female respondents and 92% of male respondents would not want to stop working with a co-worker, despite receiving the information regarding their mental illness—*p* < 0.001. Almost one-third (33%) of non-physician medical students believe that psychiatric patients will never fully recover, compared to 18% of future physicians (*p* < 0.01). A detailed comparison of MICA-2 responses in relation to sex, a completed psychiatry course, experience with mental illness, and a major is shown in Table 5.

The analysis of potential confounding factors is shown in Table 6.

## 4. Discussion 

The presented study concerned the assessment of the stigmatisation of psychiatry and its patients by students at Polish medical schools. A particular emphasis was put on sex, completing a course in psychiatry, and having personal experience with mental problems. The mean MICA-2 score of 41.05 was higher in Polish students than in students from Spain (38.16) [15] and the Czech Republic (40.11) [16]. In contrast, students from Poland achieved lower scores than students from the U.S. state of Georgia (68.44) [17], Egypt (42.67) [12], Thailand (43.16) or India (46.56) [18]. However, it should be noted that this study is not representative of the group of Polish students of medical universities. The data obtained from this study are consistent with global reports [15,16,17,18], according to which men showed significantly higher levels of stigmatisation than women. This may be related to the fact that men are more likely to perceive mental disorders as a weakness of character, while women are more likely to associate the said disorders with traumatic experiences [19]. Moreover, individual experience with mental disorders was confirmed to reduce the level of stigmatisation of mental health problems [12,15,16,17,18].

The role of proper education is crucial in reducing the level of stigma among medical students [20]. This is why it is concerning that neither the stage of tertiary education nor the completion of the course in psychiatry had a significant effect on the final score of the questionnaire. Medical major students achieved significantly lower scores than students of other fields of medicine (40.09 vs. 42.56; *p* < 0.001). The reason for this disparity may be related to the greater time frame of classes, particularly in the face-to-face interaction with psychiatric patients in facilities related to mental health. Furthermore, it is useful to consistently practice the psychiatric history during clinical classes, as a significant number of respondents feel uncomfortable talking to someone with mental illness. This is particularly important as part of the training of future physicians of other specialisations who exhibit significantly higher levels of stigmatisation towards psychiatric patients than psychiatrists. Confrontation with psychiatric patients, who often visit university clinics in exacerbation states, is a strong emotional experience for students. If not preceded by appropriate education, clinical activities may not reduce stigma. Unfortunately, there are no direct anti-discrimination issues in the Polish psychiatry curriculum. According to one survey, which also included interns, found that they are more likely than medical students to say that mental illness is recurrent and that patients are prone to crime, which is not true [20].

In another study, general practitioners scored 44 points on average on the MICA-2 scale, while forensic psychiatry specialists scored 38 points and mental health care personnel 34.1 points [21]. In another survey, it was found that for students who declared their intent to achieve the specialisation in psychiatry, the MICA-2 score is significantly lower than that of students preferring other specialisations [22].

There are many reports concerning the beneficial effects of diversifying both the psychiatry course at medical schools and educational training on the reduction in stigmatisation. A decrease in MICA-2 scores from 48.2 to 43.5 points was observed in Australian students of medicine after 8-week psychiatry classes enriched with visits to both a health centre for individuals addicted to psychiatric substances and a geriatric clinic [10]. On the other hand, anti-discrimination training among British students of medicine had only a temporary effect on the improvement of MICA-2 scores, verified after 6 months [23]. A new training model for personnel working in the mental health field was implemented in a study conducted in China. The positive change was evident in both the scope of knowledge of mental health and a reduction in the level of negative attitudes towards the mentally ill. Moreover, the said change was sustained over time. The new model included more hours devoted to working with patients, providing training on health education by trainees, rehabilitation, and the long-term follow-up of patients, whereas the traditional course format focused mainly on clinical aspects of psychiatry [24]. The inclusion of elements of work and social education into the core psychiatry curriculum may be a promising factor contributing to the reduction in the stigmatisation of mental illness among students at medical schools. The reason is that it offers the opportunity to better understand patients’ needs and monitor the treatment process from a broader, all-inclusive perspective.

An optimistic sign is observations among future medical workers that psychiatry is widely considered to be equivalent to other fields of medicine and psychiatrists are considered to be “real physicians”. Furthermore, the use of pejorative terms towards patients, such as “nut” and “madman”, is viewed in a definitively negative way. In addition, the respondents’ answers indicate that the mentally ill are not excluded from social or professional groups. On the other hand, most respondents mistakenly view the mentally ill as more dangerous than the general population. Unfortunately, some respondents may overestimate the importance of psychosomatic aspects among the mentally ill and compromise their health due to neglecting somatic problems [25].

A significant statistical difference in MICA-2 mean scale values (42.47 vs. 39.86) was observed in the comparative analysis of the 2017 and 2020 survey groups. This relationship may result from various social campaigns related to anti-discrimination psychoeducation that are increasingly appearing in the public space, mainly in the context of depression [26]. This is especially true during the global COVID-19 pandemic, when an increasing part of the population is struggling with mental illness. This increase was also clearly demonstrated in the presented study, with as much as an 11% increase in the number of respondents who had personally experienced mental illness or observed it in their loved one [27,28]. However, a considerable cautiousness is required for final conclusions, as the second-round survey was not conducted on the same group of respondents. There is a need for further follow-up studies.

The authors are aware of the limitations of this study, which undoubtedly include the methodology of data collection using networks. However, given the current epidemiological situation prevailing in the country, online surveys are a safe and easily accessible form of study to reach the largest possible number of participants. On the other hand, there is no possibility to verify the data veracity, even if it is the respondents’ personal data, the estimation of the number of recipients who obtained the information about the survey, and the estimation of started and uncompleted survey sheets. An undoubted strength is the originality of the selected psychometric tool used for assessing both the level of stigmatisation of psychiatric patients and psychiatry as a medical field by students in Poland. It should also be noted that the group presented in this study is not representative of Polish medical students. The significant disparity between men and women should also be noted. Nevertheless, when analysing the data published by the Statistics Poland in 2019, there were 74.50% female students at medical schools and 25.5% male students, while in the presented study, the percentages are very similar—81% and 19%, respectively [29]. Additionally, the distribution of students of individual faculties differs from the actual distribution. For example, in official data from 2017, the percentage of medical students was 26%. At the time of writing this manuscript, there were no official data available for 2020. [30].

In recent decades, psychiatry has changed its image significantly. Certainly, many effective efforts have been made to destigmatise both this field of medicine and its patients. Despite this fact, the level of stigmatisation of psychiatric patients in Polish society is still rated as high. This is especially strongly expressed in economic aspects and the sense of visual and intellectual otherness [31].

The study of student groups and young physicians is of particular importance in the context of future changes in public perception of psychiatry. The reason is that the aforementioned two groups are those who will influence the mental health care system and create the image of psychiatry in patients in the near future. Meanwhile, it turns out that some harmful stereotypes still persist among Polish students at medical schools. In comparison with studies from other countries, the result can be assessed as moderate, although this does not alter the fact that it is still unsatisfactory. Undoubtedly, the psychiatry curriculum at medical schools and universities should put more emphasis on both understanding the problems faced by patients and the mental health care system. It is possible that students need better substantive preparation before their first clinical contact with patients, as the emotions aroused by the otherness of psychiatric patients compared to patients from other clinical areas may reinforce false beliefs and distort perception. Currently, a mental recovery assistant is being introduced in Poland. These are people who have survived a mental health crisis and received training to help others in need. An interesting solution would be meetings of such people with medical students. The combination of their experience and knowledge could let them effectively teach the attitude towards a psychiatric patient and reduce stigma [32]. The teacher has a huge influence on creating the attitudes of medical students, which is why complementary training in psychiatry would be worth considering for senior doctors trained in the prior system. It is apparent that their attitude to the subject of psychiatry based on obsolete knowledge may discourage younger adepts of medicine from choosing a specialization such as psychiatry [20]. Currently, psychiatry in Poland is being deeply remodelled. Service provision predominantly based on mental facilities with several hospital beds is still functioning in the country. Education and training in such a model do not cover the needs of students and rather support inequity. In the last two years, a new programme has been introduced with the strong support of the government aiming at the shift of mental services towards social psychiatry. Twenty-three centres of mental health were established across the country, each with a wide range of novel interventions, prevention programs, and supportive actions for mentally ill persons promoting their social inclusion. The authors strongly believe that such a model of treatment will benefit from better training for students and young doctors in the atmosphere of understanding, support, and humanity. The pilot program will end in December 2022, and the third stage of research in the field of stigma among students is planned [33].

## 5. Conclusions

Women are more favourably disposed to psychiatry and psychiatric patients than men. As there is a high level of stigmatisation, strategies need to be implemented to improve the quality of education and increase direct student–patient contact. Although there has been a significant change in the attitude towards both psychiatric patients and psychiatry over the years, a high level of stigmatisation can still be observed, and there is a need for a further increase in mental health awareness among young medical workers.

## Figures and Tables

**Table 1:** Characteristics of the study group.

	Total Amount		First-Round Survey—2017		Second-Round Survey—2020	
Sex						
Female	857	(81%)	386	(80%)	471	(82%)
Male	196	(19%)	94	(20%)	102	(22%)
Major						
Medicine	645	(61%)	320	(67%)	325	(57%)
Other	408	(39%)	160	(33%)	248	(43%)
Year of studies						
I	137	(13%)	32	(7%)	105	(18%)
II	177	(17%)	55	(11%)	122	(21%)
III	196	(19%)	108	(23%)	88	(16%)
IV	167	(16%)	99	(20%)	68	(12%)
V	196	(19%)	81	(17%)	115	(20%)
VI	122	(11%)	70	(15%)	52	(9%)
Internship	58	(5%)	35	(7%)	23	(4%)
Mental health problems						
Yes, me	525	(50%)	228	(48%)	297	(52%)
Yes, a person close to me	296	(28%)	116	(24%)	180	(31%)
No	232	(22%)	136	(28%)	96	(17%)
Psychiatry classes undertaken						
Yes	523	(50%)	276	(57%)	247	(43%)
No	530	(50%)	204	(43%)	326	(57%)

**Table 2:** Mean MICA-2 score.

Variable		Mean Score (SD)	*p*
**Sex**	Female	40.39 ± 7.59	<0.001
	Male	43.94 ± 9.44	
**Major**	Medicine	40.09 ± 8.38	<0.001
	Other	42.56 ± 7.36	
**Psychiatry classes undertaken**	Yes	41.35 ± 8.28	0.105
	No	40.76 ± 7.90	
**Mental health problems**	Yes, me	40.48 ± 7.37	<0.001
	Yes, a person close to me	40.03 ± 8.34	
	No	44.10 ± 7.65	

**Table 3:** The comparison of MICA-2 in relation to the major.

	Medicine		Other Majors		*p*
**Sex**					
Female	39.23 ± 7.70	(n = 496)	41.98 ± 7.15	(n = 361)	<0.001
Male	42.96 ± 9.82	(n = 149)	47.06 ± 7.37	(n = 47)	0.002
**Psychiatry classes undertaken**					
Yes	40.01 ± 8.57	(n = 321)	43.46 ± 7.30	(n = 203)	<0.001
No	40.17 ± 8.19	(n = 324)	41.67 ± 7.32	(n = 205)	0.013
**Mental health problems**					
Yes, me	38.93 ± 8.31	(n = 331)	41.90 ± 8.06	(n = 194)	<0.001
Yes, a person close to me	39.59 ± 7.91	(n = 178)	41.83 ± 6.25	(n = 117)	0.005
No	43.60 ± 8.22	(n = 135)	44.77 ± 6.71	(n = 97)	0.327

**Table 4:** The analysis of individual questions addressed in the MICA-2 questionnaire.

Question	I Strongly Disagree	I Disagree	I Rather Disagree	I Rather Agree	I Agree	I Strongly Agree
1 I just learn about psychiatry because it is in the exam and would not bother reading additional material on it.	24%	33%	10%	14%	13%	6%
2. People with a severe mental illness can never recover enough to have a good quality of life.	22%	42%	12%	15%	7%	2%
3. Psychiatry is just as scientific as other fields of medicine.	1%	1%	1%	3%	23%	71%
4. If I had a mental illness, I would never admit this to any of my friends because I would fear being treated differently.	20%	36%	13%	22%	7%	2%
5. People with a severe mental illness are dangerous more often than not.	7%	27%	15%	37%	11%	3%
6. Psychiatrists know more about the lives of people treated for a mental illness than do family members or friends.	2%	13%	14%	35%	28%	8%
7. If I had a mental illness, I would never admit this to my colleagues for fear of being treated differently.	12%	31%	12%	26%	14%	5%
8. Being a psychiatrist is not like being a real doctor.	72%	24%	1%	2%	0.50%	0.50%
9. If a consultant psychiatrist instructed me to treat people with a mental illness in a disrespectful manner, I would not follow their instructions	5%	7%	2%	4%	27%	55%
10. I feel as comfortable talking to a person with a mental illness as I do talking to a person with a physical illness.	3%	19%	21%	22%	25%	10%
11. It is important that any doctor supporting a person with a mental illness also assesses their physical health.	0.10%	0.40%	0.50%	6%	45%	48%
12. The public does not need to be protected from people with a severe mental illness.	5%	22%	32%	21%	17%	3%
13. If a person with a mental illness complained of physical symptoms (such as chest pain), I would attribute it to their mental illness.	6%	39%	21%	28%	5.50%	0.50%
14. General practitioners should not be expected to complete a thorough assessment for people with psychiatric symptoms because they can be referred to a psychiatrist.	13%	34%	16%	16%	17%	4%
15. I would use the terms ‘crazy’, ‘nutter’, ‘mad’, etc. to describe people with a mental illness who I have seen in my work	51%	34%	6%	5%	3%	1%
16. If a colleague told me they had a mental illness, I would still want to work with them	6%	13%	14%	10%	33%	24%

**Table 5:** The percentage of positive responses (“I strongly agree”, “I agree”, and “I rather agree”) to each question addressed in the MICA-2 scale.

Question	Sex			Psychiatrist			Psychiatric Disorders			Major		
	Female	Male	*p*	Yes	No	*p*	Yes	No	*p*	Medicine	Other	*p*
1. I studied psychiatry only because I had an exam and I did not read any additional related materials.	30	39	**0.02**	46	19	**<0.001**	27	53	**<0.001**	27	41	**<0.001**
2. The seriously mentally ill will never recover enough to achieve a satisfying quality of life.	23	29	0.114	26	22	0.136	23	30	**0.016**	18	33	**<0.001**
3. Psychiatry is part of science just like other fields of medicine.	98	94	**0.014**	97	97	0.559	97	97	0.606	97	98	0.451
4. If I suffered from mental illness, I would never tell any of my friends about it because I would be afraid that they would treat me differently.	32	32	0.926	35	29	**0.033**	29	41	**<0.001**	31	34	0.231
5. The seriously mentally ill often pose a threat to others.	50	55	0.202	47	55	**0.011**	48	61	**<0.001**	47	58	**<0.001**
6. Psychiatrists know more about the life of patients being treated for mental illness than family members of those people.	72	70	0.729	71	71	0.946	71	73	<0.477	70	73	0.353
7. If I suffered from mental illness, I would never tell any of my friends about it for fear that they would treat me differently.	45	40	0.213	44	43	0.8	41	53	**0.001**	43	45	0.416
8. A psychiatrist is not a real doctor.	2	7	**<0.001**	4	2	**0.033**	3	3	0.83	4	3	0.95
9. If a psychiatrist supervisor instructed me to treat a mentally ill person disrespectfully, I would not follow that instruction.	88	78	**<0.001**	86	86	0.791	87	84	0.249	87	84	0.163
10. I feel equally comfortable talking to a mentally ill person as I am talking to a physically ill person.	59	49	**0.011**	56	59	0.262	61	46	**<0.001**	55	62	**0.014**
11. It is important that any physician supporting the mentally ill person should also assess their physical health.	99	99	0.357	99	99	0.981	99	99	0.803	99	99	0.834
12. Society does not need to be protected from the seriously mentally ill.	44	30	**<0.001**	43	40	0.351	44	32	**0.001**	42	41	0.804
13. If the mentally ill person complained of physical ailments (e.g., chest pain), I would link it to their mental illness.	33	40	0.049	29	39	**0.001**	35	31	0.181	28	43	**<0.001**
14. General practitioners should not be expected to perform a comprehensive health assessment of individuals with psychiatric symptoms because they may be referred to psychiatrists.	35	46	**0.003**	30	42	**<0.001**	36	40	0.299	34	41	**0.005**
15. I would use the terms “mad”, “madman”, “nut” to describe the mentally ill I have met in my work.	7	18	**<0.001**	11	8	0.123	9	13	0.077	10	9	0.706
16. If my coworker told me that they suffered from mental illness I would still want to work with them.	96	93	0.028	95	96	0.615	95	97	0.15	96	95	0.624

Significant effects (*p* < 0.05) are marked in bold.

**Table 6:** Analysis of potential confounding factors.

	F-Value	*p*-Value
Sex	4.37	0.036
Major	0.61	0.652
Year of studies	1.12	0.351
Psychiatry classes undertaken	0.02	0.901
Mental health problems	1.86	0.156

## Data Availability

The data presented in this study are available on request from the corresponding author.
